# Antithrombotic use in retinal artery occlusion: A narrative review

**DOI:** 10.1002/kjm2.12938

**Published:** 2025-01-16

**Authors:** Wan‐Yu Liu, Yun‐Hsin Tang, Yi‐Hsing Chen

**Affiliations:** ^1^ Department of Medical Education Chang Gung Memorial Hospital, Linkou Main Branch Taoyuan Taiwan; ^2^ Department of Obstetrics and Gynecology Chang Gung Memorial Hospital, Linkou Main Branch Taoyuan Taiwan; ^3^ College of Medicine Chang Gung University Taoyuan Taiwan; ^4^ Department of Ophthalmology Chang Gung Memorial Hospital, Linkou Main Branch Taoyuan Taiwan

**Keywords:** anticoagulant therapy, antiplatelet therapy, retinal artery occlusion

## Abstract

Retinal artery occlusion (RAO) is a critical ophthalmic emergency with a high risk of significant visual impairment. While traditional treatment aims to promptly restore blood flow to the retina, recent research has investigated the potential benefits of anticoagulation therapy for managing this condition. This paper reviews current literature and clinical trials investigating the efficacy and safety of anticoagulant and antiplatelet therapies, such as systemic heparinization and direct oral anticoagulants and aspirin, in treating RAO. The mechanism of action involves preventing thrombus propagation and platelet aggregation to promote microvascular circulation, potentially mitigating ischemic damage and improving visual outcomes. However, controversies exist regarding the optimal timing, duration, and selection of antithrombotic agents due to the risk of hemorrhagic complications. Further large‐scale prospective studies are warranted to establish evidence‐based guidelines for incorporating antithrombotic into the standard management of RAO. This paper underscores the evolving landscape of antithrombotic therapy as a promising adjunctive treatment strategy in the management of retinal artery occlusion.

## 1 INTRODUCTION

**FIGURE 1:**
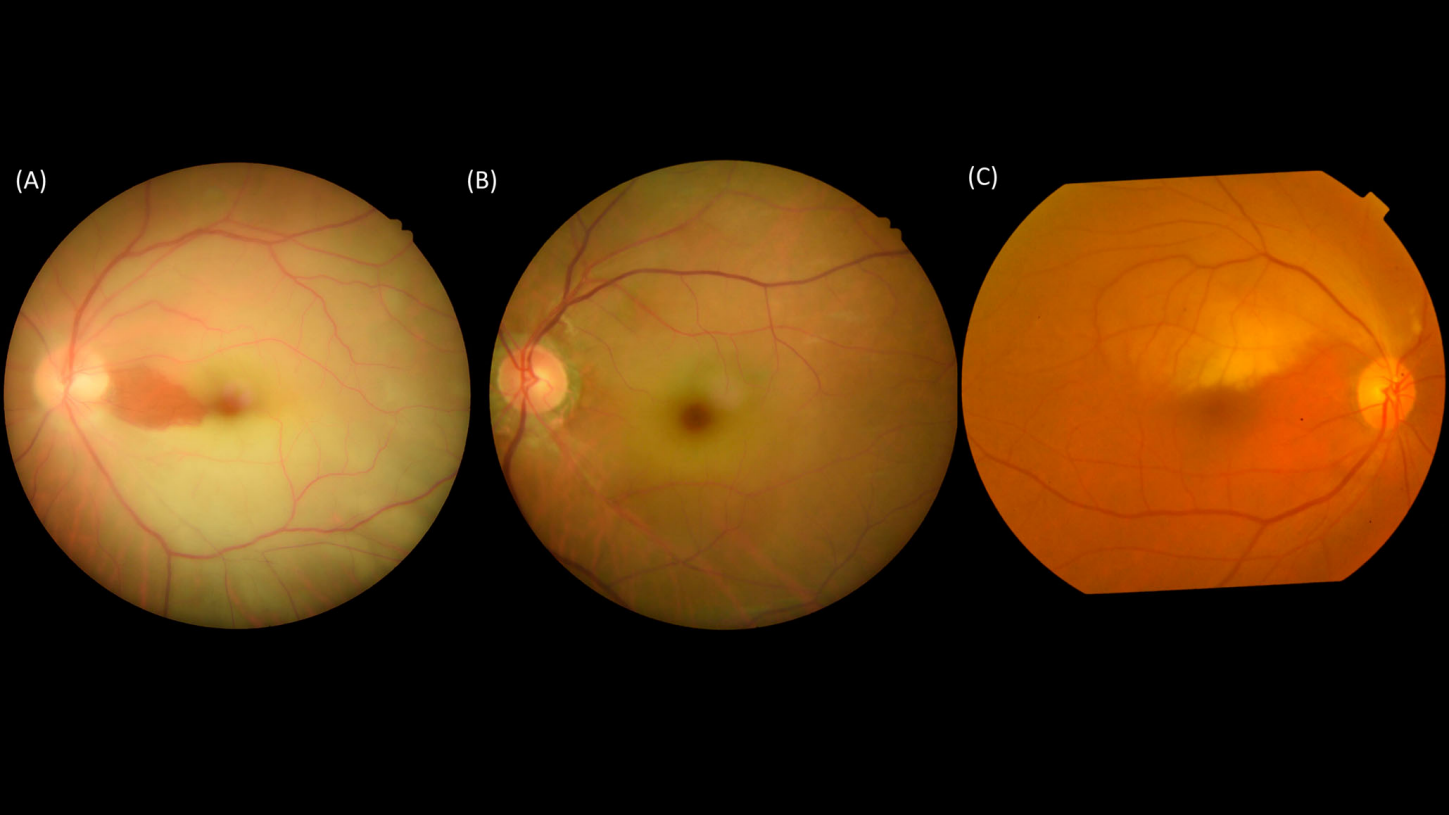
Different subtypes of retinal artery occlusion (RAO). (A) Central retinal artery occlusion (CRAO) with cilioretinal artery sparing, which preserves the blood flow from posterior ciliary circulation to supply of macula and serves as a cofounder of visual prognosis in CRAO. (B) CRAO with cilioretinal artery involved. (C) Branch retinal artery occlusion (BRAO) which involves superior temporal branch of retinal artery.

Retinal artery occlusion (RAO) is a critical illness as it causes sudden, painless, and often disastrous vision loss which is often permanent (Figure 1). The incidence of RAO has been reported 1.0–2.5 per 100,000 person‐years and does not differ between the western and Asian population.^1^, ^2^, ^3^ The incidence is increased with aging and has been reported up to 57 per 100,000 person‐years at the age group of 80–84 years in a German study.^4^ More than 80% of central retinal artery occlusion (CRAO) patients present with poor initial best corrected visual acuity (BCVA) less than 20/200 and 77% patient will remain BCVA less than 20/200.^5^ Besides, 22% of non‐arteritic CRAO patients experienced BCVA improvement within the first 7 days of onset and only 10% of patients had any appreciable improvement thereafter in the natural course study.^5^ Embolism is the most common etiology of RAO, typically originating from the carotid artery and heart valves, composing of cholesterol, calcific material or platelet‐fibrin plugs.^6^, ^7^ The lateral two components have been indicated for poor visual outcome in RAO patients.^8^

The risk factors for RAO incidence have been reported including hypertension, diabetes mellitus, dyslipidemia, atrial fibrillation (AFib), and previous stroke.^9^ Thrombophilia risk factors such as low protein C level, low free protein S level, hyperhomocysteinemia (>5 μmol/L), and factor V Leiden mutation were significantly associated with RAO.^10^ The relationship between AFib, stroke and RAO attracted attention recently. Patients with RAO have an increased risk of stroke and AFib, with a 2.2‐fold higher risk of stroke and a 1.5‐fold higher risk of AFib.^11^, ^12^ A study utilizing Taiwan's National Health Insurance Database revealed that AFib and coronary artery disease (CAD) independently increase risk of RAO, with hazard ratios of 8.0 and 5.3, respectively.^13^ This suggest that both AFib and CAD serve as risk factors and the possible source of emboli for RAO. Hence, AFib serves as a common risk factor for RAO and embolic stroke, indicating that they may share similar pathogenesis and management approach.

Managements strategies have been used to treat RAO, including anterior chamber paracentesis, ocular massage, intraocular pressure lowering drugs, and hyperbaric oxygen (HBO). However, no randomized controlled trials have demonstrated that these have a better outcome when compared to natural history.^14^ As RAO presents as a vascular ischemia event and it has been reported to be associated with increasing incidence of ischemic stroke, the clinical management has recently been suggested to follow the stroke protocol.^15^ Small pilot studies indicated that intra‐arterial therapy was superior to other conservative treatments for BCVA outcomes if administered within 6 h of RAO onset.^16^, ^17^, ^18^ The reports for the efficacy of anticoagulant and antiplatelet agents in RAO patients in the long term are limited.

This article aims to discuss the current management of RAO and provides a narrative review on the role of anticoagulant and antiplatelet treatment in RAO.

## 2 MATERIALS AND METHODS

For this review, we used databases such as PubMed and EMBASE to find the publications till 2023 August. Search terms used included “retinal artery occlusion,” in combination with “antiplatelet agents,” “Platelet aggregation inhibitors,” “Aspirin,” “clopidogrel,” “dipyridamole,” “ticagrelor,” “ticlopidine,” “tirofiban,” “eptifibatide,” “abciximab,” for searching related articles. As for anticoagulant agents, search terms included “retinal artery occlusion,” in combination with “anticoagulant agents,” “heparin,” “fondaparinux,” “enoxaparin,” “warfarin,” “direct thrombin inhibitor,” “blood clotting factor Xa inhibitor,” for searching related articles. Keywords were combined using appropriate Boolean operators. In addition, the reference lists of all articles identified during the database search were examined to identify other potentially relevant articles. We included patients with any type of RAO who were treated with antiplatelet or anticoagulant medication after symptom onset and reported changes in vision or visual field. Patients meeting these criteria but had other eye diseases making it difficult to distinguish the effects of antithrombotic therapy, were excluded. The following data were extracted from the included studies: authors, year of publication, patients' demographic information (age, sex, medical history), treatment options, treatment duration, and vision outcomes.

## 3 RESULTS

### 3.1 Literature search

**FIGURE 2:**
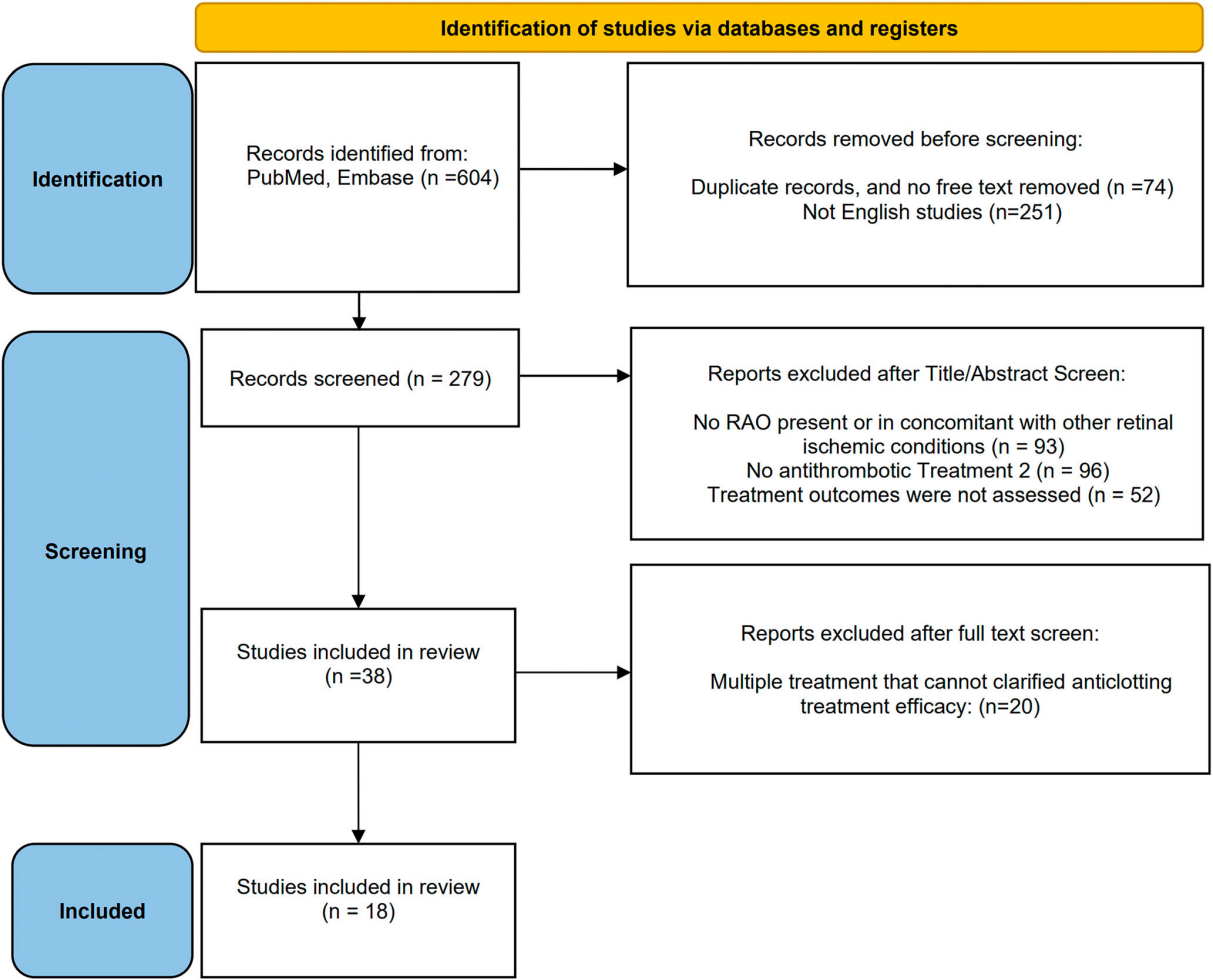
PRISMA 2020 flow diagram outlining the selection process for the inclusion of the studies in the systematic review.

As shown in Figure 2, 193 and 411 articles were enrolled in PubMed and EMBASE databases respectively. There were 74 studies which were duplicated or not available in full text; 251 studies were not in English text form; 93 studies were excluded due to other underlying systemic disease relevant to RAO (giant cell arteritis, antiphospholipid syndrome, Susac syndrome, or other hypercoagulable disease) but not currently diagnosed with RAO or presented with concomitant other retinal ischemic conditions (combined retinal vein occlusion, or ischemic optic neuropathy); 96 studies were excluded due to including RAO patients who received no antithrombotic treatment; 52 studies were excluded due to unobtainable ocular efficacy of antithrombotic treatment. Finally, 20 studies were excluded due to multiple therapies administered, and the efficacy of antithrombotic treatment was not clear. Finally, this review included 8 articles from Pubmed and 10 from EMBASE.

### 3.2 Patient characteristics

The study included 16 case reports and 2 retrospective cohort studies (Table 1).^19^, ^20^, ^21^, ^22^, ^23^, ^24^, ^25^, ^26^, ^27^, ^28^, ^29^, ^30^, ^31^, ^32^, ^33^, ^34^, ^35^, ^36^ Eleven articles were focused on CRAO patients and the remainder on BRAO patients. The age range of the patients was from 6 to 78 years old. Most of the patients had associated systemic conditions which were related to hypercoagulable status or thrombophilia conditions, such as anti‐phospholipid syndrome, carotid artery stenosis, or homocysteinemia and so on.

**TABLE 1:** Studies involving antithrombotic treatment for retinal artery occlusion.

	Study type	RAO type	Age, gender	Antithrombotic treatment	Duration of Tx	Associated disease	Initial BCVA	Final BCVA	VF recovery
Stanescu et al.^19^	Case report (*N* = 1)	Hemi‐CRAO	64, F	Aspirin	N/A	Giant cell arteritis	6/12	N/A	No
Baciu et al.^20^	Case report (*N* = 1)	BRAO	21, M	Aspirin	5 months	Livedo reticularis	20/80	20/25	N/A
Shirato et al.^21^	Case report (*N* = 1)	CRAO	75, F	Aspirin	4 months	Carotid artery stenosis	20/100	20/63	N/A
Chatziralli et al.^22^	Case report (*N* = 1)	BRAO	35, M	Aspirin and clopidogrel	15 days	Patent foramen ovale	6/12	6/7.5	N/A
Pierro et al.^23^	Case report (*N* = 1)	CRAO	65, F	Tirofiban	14 h	After internal carotid stent replacement	HM	20/20	N/A
Hölschermann et al.^24^	Retrospective study (*N* = 18)	CRAO	46–78, (F = 3)	Tirofiban	>48 h	Dyslipidemia, HTN	NLP ~ 0.05	2/3 improved (>2 lines). 20/20 (*N* = 2)	N/A
Riccardo et al.^25^	Case report (*N* = 1)	BRAO	34, M	Enoxaparin	4 weeks	After orbital cavernous hemangioma excision	CF	9/10	Partial
Bruscolini et al.^26^	Case report (*N* = 1)	BRAO	27, M	Enoxaparin	4 weeks	Gaucher disease	20/25	20/20	Partial
Turkistani et al.^27^	Case report (*N* = 1)	CRAO	34, F	Heparin	N/A	Ovarian hyperstimulation syndrome	HM	HM	No
Hua et al.^28^	Case report (*N* = 1)	CRAO	20, F	Heparin	3 days	SLE, DVT	CF/5 ft	OD: 20/800 OS: 20/400	N/A
Singh et al.^29^	Case report (*N* = 1)	CRAO	72, F	Warfarin	N/A	Antiphospholipid syndrome	NLP	NLP	No
Zahid et al.^30^	Case report (*N* = 1)	CRAO	14, F	Rivaroxaban	2 months	Homocystinuria	NLP	6/9	N/A
Saxonhouse et al.^31^	Case report (*N* = 1)	BRAO	15, M	LMWH followed by warfarin	4 weeks	Antiphospholipid syndrome	20/30	20/30	Partial
Agrawal et al.^32^	Case report (N = 1)	CRAO	10, M	LMWH 1 week followed by warfarin	4 weeks	Nephrotic syndrome	NLP	6/60	N/A
Abbati et al.^33^	Case report (*N* = 1)	CRAO with COVID	6, F	LMWH then aspirin	5 months	Nil	OS: HM 30 cm OD: NLP	OS: CF 30 cm OD: NLP	N/A
Nishiyama et al.^36^	Case report (*N* = 1)	CRAO	53, M	Argatroban (iv) then heparin (iv)	2 weeks	Asthma	20/200	20/28	N/A
Jinghua et al.^34^	Case report (*N* = 1)	CRAO after COVID vaccination	40, F	Fondaparinux and aspirin	2 weeks	HTN, Grave's disease	NLP	NLP	No
John et al.^35^	Retrospective study (*N* = 17)	RAO	51 (mean) (F = 10)	Aspirin/warfarin/LMWH	6–12 months	53% had thrombophilia factor	30%: complete recovery 28%: partial recovery		N/A

Abbreviations: BRAO, branch retinal artery occlusion; CF, counting finger; CRAO, central retinal artery occlusion; F, female; HM, hand motion; HTN, hypertension; LMWH, low molecular weight heparin; M, male; N/A, not available; NLP, no light perception; Tx, therapy; VF, visual field.

### 3.3 Treatment and outcomes of antithrombotic treatment

Among antiplatelet treatments for RAO, aspirin was the most used medication, followed by tirofiban and clopidogrel.^19^, ^20^, ^21^, ^22^, ^23^, ^24^, ^33^, ^34^, ^35^ Two retrospective studies involved tirofiban and aspirin for RAO and the results of visual improvement were variable (Table 1). Out of seven case reports, 30% demonstrated either no improvement or a decline in vision with antiplatelet therapy in RAO.

There were 11 case reports investigating the efficacy of anticoagulant therapy for treating RAO, which demonstrated variable in BCVA outcome.^25^, ^26^, ^27^, ^28^, ^29^, ^30^, ^31^, ^32^, ^33^, ^34^, ^36^ The adopted anticoagulants being reported include indirect anticoagulants such as heparin, low molecular weight heparin (LMWH), and enoxaparin; vitamin K antagonist (warfarin); selective direct thrombin inhibitor (Argatroban, Texas Biotechnology Corporation; Smith‐Kline Beecham Pharmaceuticals), indirect Xa inhibitor (Fondaparinux, Arixtra®, GlaxoSmithKline), and direct Xa inhibitor (Rivaroxaban). Among these, five studies have employed combined antithrombotic therapy.^31^, ^32^, ^33^, ^34^, ^36^ In case reports utilizing indirect anticoagulants, 7 out of the 10 patients displayed adverse effects of coagulopathy.^26^, ^27^, ^28^, ^29^, ^31^, ^34^, ^37^ With regards to BCVA outcomes, in seven cases (63.6%), there was an improvement in BCVA, while in one patient (9.1%), visual acuity did not improve but there was partial improvement in visual fields.

## 4 DISCUSSION

### 4.1 Current management in RAO

Conventional treatments for RAO patients include anterior chamber paracentesis, ocular massage, intraocular pressure lowering drugs, hyperbaric oxygen (HBO), neodymium‐doped yttrium aluminum garnet (Nd:YAG) laser embolysis, intravascular thrombolysis and antithrombotic therapy. The goal is to restore retinal circulation and reduce irreversible damage to the retina.

### 4.2 Conservative management for RAO

In a meta‐analysis published in 2022, inhalation of 95% oxygen and 5% carbon dioxide (HBO) therapy, which aims to increase oxygen levels to ischemic area to achieve neuroprotection, does not significantly improve the final visual outcome in RAO patients.^38^ Ocular massage and intraocular pressure lowering drugs, which attempt to create intraocular pressure fluctuations to dislodge the emboli, were reported as not beneficial as sole therapy in the literature.^39^ Although there were some studies evaluating these therapies in conjunction with others, the evidence of benefit remains uncertain.^40^, ^41^ Another treatment modality of anterior chamber paracentesis has not demonstrated significant improvement in BCVA in patients who received such treatment within 6 h of onset compared to those without, disputing its role for management of RAO.^42^ Recent meta‐analysis on Nd:YAG laser embolysis demonstrated significant improvement of visual acuity, but several issues persist: 71% patients were BRAO, which has a better prognosis anyway, potentially skewing the results. In addition, safety concerns remain, with 54% of patients experiencing vitreous or pre‐retinal hemorrhage.^43^ Furthermore, only 21% of RAO patients present visible emboli that were candidate for this treatment.^44^ These factors therefore limit the feasibility and safety of Nd:YAG laser treatment.

### 4.3 Intravascular thrombolysis for RAO

Intravascular thrombolysis aims to dissolve the occluding platelet‐fibrin plugs, the concept of this being similar to that used in treating ischemic strokes. Numerous retrospective studies demonstrated the efficacy of intra‐arterial thrombolysis in comparison to conservative treatments for CRAO.^45^, ^46^, ^47^ A retrospective study of 28 RAO patients who received intra‐arterial thrombolysis at a mean duration of 6.5 h after symptom onset illustrated a significant BCVA improvement of more than three lines at mean follow‐up of 2.2 months as compared to controls.^48^ In another prospective randomized controlled trial published in 2010, when the RAO developed within an average of 11 h and patients receiving intra‐arterial thrombolysis were analyzed and demonstrated a significant visual improvement after 1 month. However, no significant difference was observed when comparing it to conservative treatment, and the intra‐arterial thrombolysis therapy demonstrated a higher rate of adverse reactions.^49^ The outcomes may be attributed to the duration from the onset of symptoms to treatment, types of RAO and different sources of emboli. In a cohort study, the administration of IV alteplase within 4.5 h in RAO patients demonstrated a significant improvement in final BCVA, comparing to both patients who did not receive fibrinolysis treatment and those who received treatment beyond 4.5 h, indicating the efficacy of fibrinolysis treatment and the importance of the treatment window.^50^ In a meta‐analysis investigating the treatment window for thrombolysis, patients who received treatment within 6 h of onset had better visual outcomes.^51^ Although there is no current strong evidence supporting the efficacy of thrombolytic therapy for treating RAO and no specific suggestions regarding the interval between treatment and symptom onset, a survey conducted among 45 teaching hospitals in USA revealed that 53% of the treatment teams expressed tendency to treat RAO with fibrinolysis.^52^

### 4.4 Role of antiplatelet drugs in RAO

In a retrospective study investigating the risk factors for thrombophilia in patients with RAO and retinal vein occlusion (RVO), it was found that approximately 53% of RAO patients had at least one thrombophilia factor, including antiphospholipid antibodies, prothrombin gene mutation, elevated homocysteine, protein C and protein S deficiencies.^35^ In addition, 56% of the studied patients being treated with aspirin, and followed up for more than 6 months, 71% manifested varying degrees of improvement in their BCVA. Furthermore, RAO often serves as an early clinical presentation in individuals predisposed to thrombophilia.^53^ For instance, in a case report concerning a pregnant woman with RAO who was treated with aspirin was in fact diagnosed with familial thrombophilia and had elevated factor VIII and decreased protein S level.^53^ In these patients RAO presented as initial sign of systemic disease, and early administration of anti‐thrombotic medications post‐RAO occurrence plays a crucial role, both in acute thrombus dissolution and in preventing future thrombus formation in other organs. Up to date, there are no antiplatelets being raised for clinical trials in the treatment of RAO. However, there are currently two ongoing randomized controlled trials with antiplatelet drugs serving as the control arm. The TenCRAOS trial (ClinicalTrials.gov Identifier: NCT04526951), which aims to enroll 78 patients with CRAO and randomly assign them into two Tenecteplase and aspirin treatment groups. Its primary outcome measurement is the proportion of patients with initial BCVA ≤0.7 logMAR (logarithm of the minimum angle of resolution) who had at least 0.3 logMAR BCVA improvement in the affected eye at 30 ± 5 days after treatment. The other is the THEIA trial (ClinicalTrials.gov Identifier: NCT03197194), which plans to enroll 70 CRAO patients and randomize them to either Alteplase or aspirin group. Its primary outcome measurement is the BCVA improvement at 1 month. Also, there were some retrospective studies and sporadic case reports indicating the benefits of antiplatelet in RAO patients which briefly listed in the Table 1. Baciu et al. reported a patient with livedo reticularis without any thrombophilia risk factors, who developed BRAO and had BCVA improvement from 20/80 to 20/25 after 5 months of aspirin treatment.^20^ Chatziralli et al. reported a patient with a patent foramen ovale, who developed BRAO and had BCVA improvement from 6/12 to 6/7.5 after 2 weeks of combined aspirin and clopidogrel therapy and was thrombotic event free after follow‐up of 9 months.^22^ Shirato et al. reported a carotid artery stenosis patient who presented with CRAO and whose vision improved from 20/100 to 20/63 after 1 month of aspirin.^21^ In a retrospective study comparing the effectiveness of various conservative treatments for patients with RAO, it was concluded that the use of antiplatelet agents such as oral acetylsalicylate was inversely correlated with final BCVA in complete CRAO patients.^54^ However, this study was complicated as 96% of the study populations had received four or more conservative treatment modalities, making it difficult to adequately assess the impact of aspirin on the treatment of RAO. In addition, the inclusion criteria for patients were limited to those with complete occlusion, which may result in poorer outcomes when compared to patients with incomplete RAO or fovea‐sparing RAO. In patients with Susac syndrome presenting with RAO, there are no current treatment guidelines. In clinical practice, it is common to administer anti‐thrombotic medications, including aspirin, when patients have an RAO,^55^, ^56^, ^57^ to inhibit thrombus formation. In 2017, a case report demonstrated that in a Susac patient who developed BRAO and received early aspirin and immunosuppressive agents' administration within 3 days resulted in a restoration of retinal artery perfusion and disappearance of Gass plaques.^57^

The risk of stroke, myocardial infraction, and death was increased following RAO, especially stroke, with an odds ratio of 50.7 compared to the general population.^58^ Current research indicates that antiplatelet therapy does not confer a protective effect in secondary prevention against stroke, myocardial infarction, and subsequent mortality in patients with RAO.^59^ Vestergaard et al. demonstrated that CRAO patients who were given aspirin had a small reduction in risk of further stroke or death for more than 1 year follow‐up duration.^58^ In addition, the CRAO group taking clopidogrel demonstrated a similar risk for further stroke compared to aspirin. Interestingly, even among individuals in the analyzed population without pre‐existing cardiovascular conditions such as ischemic heart disease or history of stroke, 1 year of aspirin treatment still significantly reduced the risk of stroke.^58^ Recent studies examining the risk of stroke in patients with RAO reported that the majority of patients developed strokes within the first 30 days after RAO onset.^11^, ^60^ Therefore, the clinical efficacy of administering aspirin to RAO patients for stroke prevention remains a subject of debate. For example, some retrospective studies suggest that aspirin had protective effects on cranial ischemic complications (including RAO) in giant cell arteritis (GCA) patients.^61^ Therefore, aspirin is commonly utilized as an adjuvant treatment to prevent thrombus formation at the distal end of inflamed blood vessels.^62^ In a series of 175 GCA patients who were on long‐term aspirin therapy there was a significantly lower incidence of ischemic ocular complication compared to those who did not receive it, especially in the first 3 months of treatment (3% vs. 13%, *p* = 0.02).^61^ In addition, for ocular and systemic safety issue concerns, no bleeding complications were observed.^63^ To date, no studies have investigated the impact of aspirin on the occurrence of RAO in patients with giant cell arteritis.^19^

In addition to aspirin, which is commonly used as a conservative management for RAO, there have been several sporadic case reports confirming affirmative effects of other antiplatelet drugs. Stanescu et al. described a 65‐year‐old female who experienced sudden vision loss due to CRAO when being treated with a stent placement for her partial thrombosed internal carotid artery aneurysm. Her vision was improved from hand motion to 20/20 the next day, after being administered with tirofiban intravenously within 10 min of onset.^23^ Another retrospective study also demonstrated the advantage in managing primary CRAO with tirofiban, in that 66% of CRAO patients had at least two lines of BCVA improvement and two patients (11%) even recovered vision to 20/20.^24^

### 4.5 Role of anticoagulant in RAO

Up to the present time, there remains no consensus on the usefulness of systemic anticoagulation for RAO. The Eagle study compared visual acuity and visual field changes between local intra‐arterial fibrinolysis combined with recombinant tissue plasminogen activator (rt‐PA) and conservative treatment for non‐arteritic CRAO patients. This study included patients with onset duration of less than 20 h and BCVA less than 20/63. Heparin (5000 IU) was used for local intra‐arterial fibrinolysis. However, the results indicated that there was no significant difference in visual acuity changes between the two groups. Moreover, the local intra‐arterial fibrinolysis group exhibited a higher proportion of adverse effects. Therefore, these studies suggested that local intra‐arterial fibrinolysis is not an appropriate treatment approach.^64^, ^65^

We know that some younger patients with RAO may exhibit a hypercoagulability tendency. Among young RAO patients with an average age of 27 years, 65% display a hypercoagulable status, with hyperhomocysteinemia being the most prevalent factor.^66^ Regarding the efficacy of anticoagulant medications in hyperhomocysteinemia, there is a recent case report demonstrating that a 14‐year‐old homocystinuria patient who had no local predisposing factors or prior thromboembolic episodes developed CRAO. Following treatment with rivaroxaban 10 mg per day for 2 months, her BCVA improved from no light perception to 6/9.^30^

**FIGURE 3:**
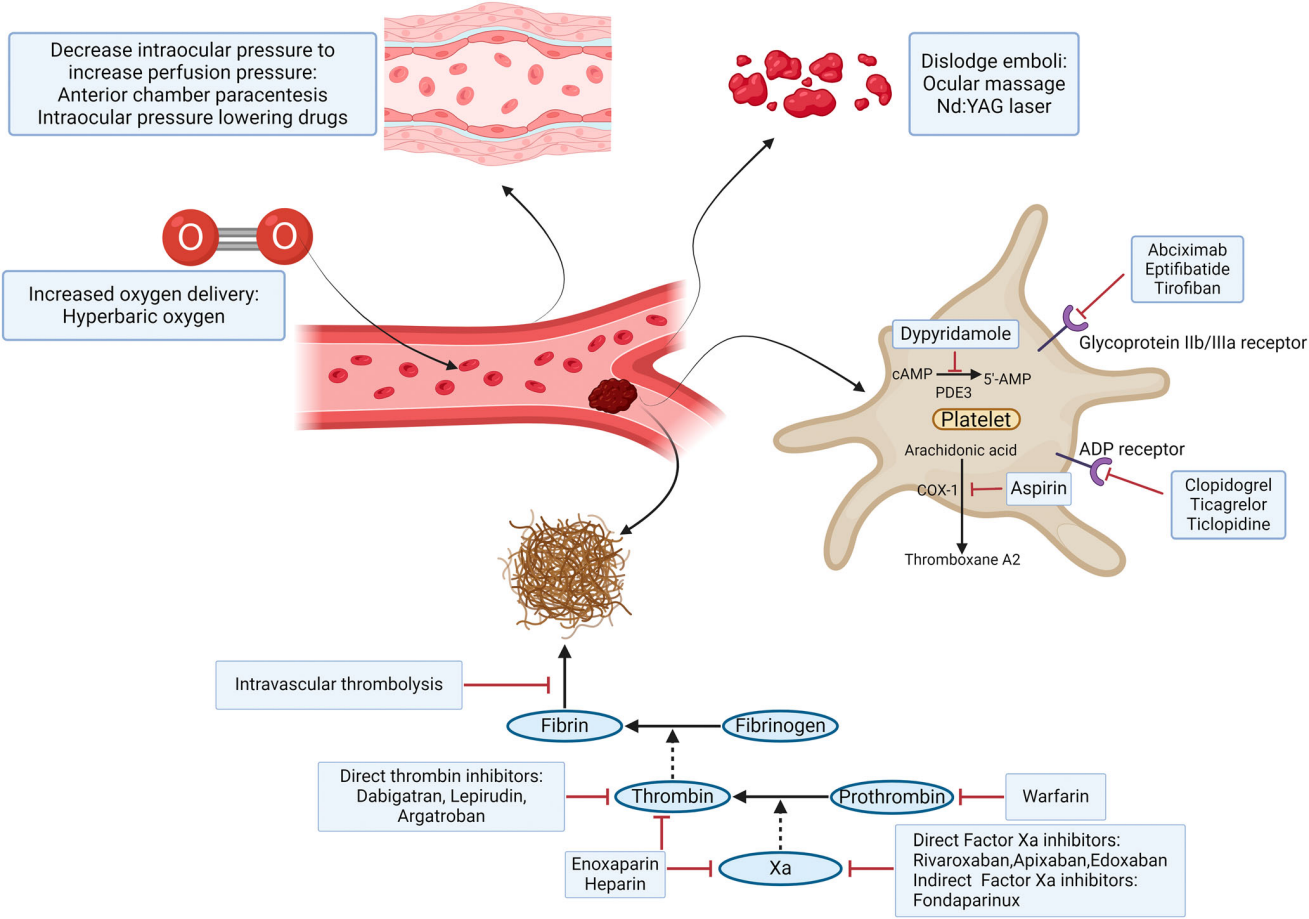
A narrative summary of mechanism and interventions for managing retinal artery occlusion in this review.

It is known that 89% of CRAO and 53% of BRAO patients may not display visible emboli (Figure 3).^67^ Regarding the effectiveness of anticoagulant therapy in patients without visible emboli, a case report of a patient with recurrent RAO was treated with combined ticlopidine and heparin (12,000 U/day) for 1 week following by heparin being replaced by warfarin for 1 month. Doppler sonography displayed decreased microembolic signals. Furthermore, no retinal ischemic events developed during the treatment period.^68^ In a study of patients with RAO without visible emboli, among those with vascular risk factors (diabetes, hypertension, dyslipidemia, AF, etc.), approximately 50% of them also had thrombophilia risk factors, such as factor V Leiden mutation or methylenetetrahydrofolate mutation, which is associated with hyperhomocysteinemia. Therefore, in RAO patients without an apparent embolic source, if they have diabetes, hypertension, dyslipidemia or other vascular risk factors, coagulation function is an important risk factor. As a result, the role of anticoagulant agents warrants further incidence for RAO.^61^, ^62^, ^63^, ^64^, ^65^, ^66^, ^67^, ^68^ In case reports using warfarin in Table 1, one out of three patients experienced an improvement in BCVA. Notably, one case report documented a CRAO patient who being treated with 2 weeks of intravenous argatroban and heparin presented a recovery of BCVA from 20/200 to 20/28.^36^ However, in a patient with CRAO who received fondaparinux followed by aspirin, the BCVA remained no light perception (NLP) after 2 weeks of treatment, which may be related to original disease severity.^34^

The pathophysiology of RAO is similar to that of cerebral stroke, with thromboembolism being one of the primary causes.^69^ In ischemic stroke, approximately 20%–40% were of unknown cause.^70^ It is now known that subclinical tachyarrhythmia and AF significantly increase the risk of CRAO by 2.5 times, comparing to those without.^71^ In a cohort study involving patients with CRAO, it was observed that the proportion of patients who developed new‐onset AF within 2 years was 49.6%. The risk was 1.64 times higher compared to the control group and similar to the risk observed in patients with cerebral stroke who developed new AF.^72^ Therefore, following a CRAO event, it is essential to focus on whether the patient has undiagnosed AF or other tachyarrhythmias. Given that CRAO is a risk factor for AF, it may be reasonable to consider preventive measures to mitigate the risk of undetected AF leading to subsequent ischemic stroke or other systemic thromboembolic events. Studies indicated that in patients with AF who experience RAO, there is a 1.39‐fold increase in thrombotic‐related events thereafter. Consequently, a history of RAO was suggested to add two points to the CHA2DS2‐VASc score, signifying that RAO is equivalent to stroke and contributes to an elevated risk of stroke.^73^ This emphasizes the need to consider the possibility of adding anticoagulants to prevent the risk of thromboembolic stroke after the occurrence of RAO. In other words, the rationale for administering anticoagulants in patients who have experienced RAO is justified.

When considering further research on anticoagulant therapy for RAO, both efficacy and side effects are critical in drug selection. A systematic review and meta‐analysis on anticoagulants for atrial fibrillation‐related stroke found that approximately 6% of patients experienced major bleeding, with 1.4% suffering intracranial bleeding and 2% gastrointestinal bleeding.^74^ Although research on anticoagulant use in RAO is limited, studies on RVO reported 4% of patients had major bleeding, including 2% with intraocular bleeding.^75^ For CRAO, Nedelmann et al.,^76^ Préterre et al.,^77^ and Schultheiss et al.^78^ indicate that one case (1%) of intracranial hemorrhage after receiving anticoagulation post‐thrombolysis. Further studies on RAO should evaluate the adverse effects, particularly intraocular bleeding, to establish safe and effective anticoagulation protocols that balance the benefits and risks.

## 5 LIMITATIONS

The literature search conducted for this paper revealed a limited number of case reports and retrospective studies on antithrombotic therapy for RAO. This may be attributed to several factors. In clinical practice, antithrombotic therapy is typically reserved for RAO patients with a high thrombotic tendency, rather than being administered to all patients. Antithrombotic therapy is frequently used as an adjunctive treatment alongside thrombolysis or other conservative managements. Consequently, there are fewer studies examining its efficacy as a standalone treatment for RAO. RAO is an acute condition that can result in severe visual impairment. Currently, there is no standardized treatment protocol for RAO patients in clinical practice, which poses challenges for conducting large‐scale study on the effectiveness of antithrombotic therapy.

## 6 CONCLUSIONS

**FIGURE 4:**
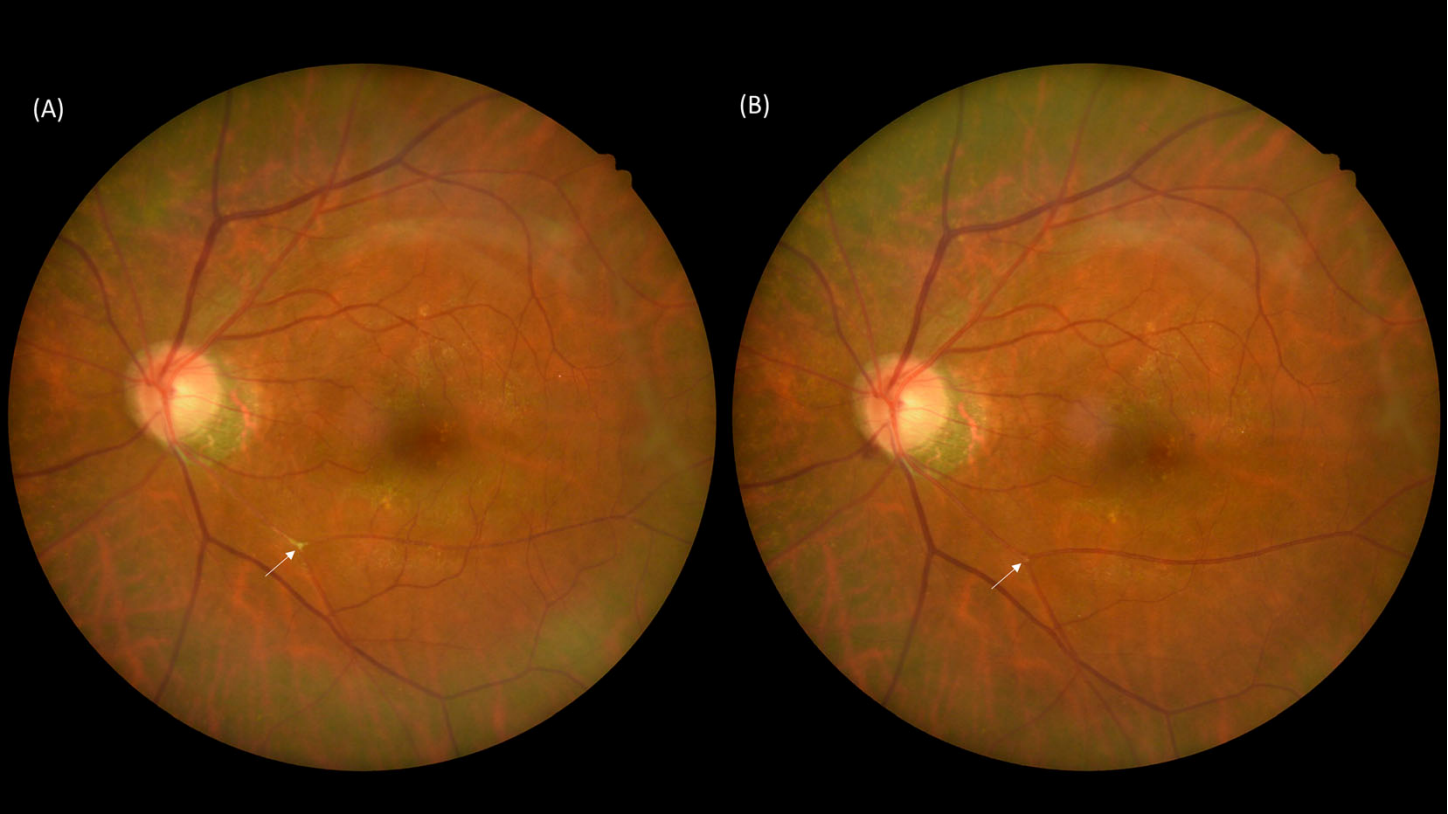
Emboli in retinal artery occlusion. (A) Color fundus photo of a patient experiencing retinal artery occlusion with embolus (white arrow) before treatment. (B) The embolus was dislodged after treatment with 2 months of rivaroxaban (white arrow).

RAO is an acute condition for which there is currently no potent treatment. This paper compiles information on the efficacy of antithrombotic drugs in the context of RAO (Figure 4). As RAO is considered an ischemic stroke and is recognized as an independent risk factor for subsequent thrombotic events in patients with AF, further research is needed to substantiate the rational and relevance of using anti‐thrombotic drugs in the case of RAO.

## CONFLICT OF INTEREST STATEMENT

The authors declare no conflict of interest.

## Data Availability

The data that support the findings of this study are available on request from the corresponding author. The data are not publicly available due to privacy or ethical restrictions.
